# Capillary Underfill Flow Simulation as a Design Tool for Flow-Optimized Encapsulation in Heterogenous Integration †

**DOI:** 10.3390/mi14101885

**Published:** 2023-09-30

**Authors:** Lisa Christin Stencel, Jörg Strogies, Bernd Müller, Rüdiger Knofe, Carsten Borwieck, Matthias Heimann

**Affiliations:** Siemens AG, T ICE ELM-DE, 13629 Berlin, Germany

**Keywords:** capillary underfill, power packaging, microchannel wicking, CFD simulation, VOF, experimental validation, void formation, predictive design tool

## Abstract

As the power electronics landscape evolves, pushing for greater vertical integration, capillary underfilling is considered a versatile encapsulation technique suited for iterative development cycles of innovative integration concepts. Since a defect-free application is critical, this study proposes a capillary two-phase flow simulation, predicting both the flow pattern and velocity with remarkable precision and efficiency. In a preliminary performance evaluation, Volume of Fluid (VOF) outperforms the Level-Set method in terms of accuracy and computation time. Strategies like HRIC blending, artificial viscosity, and implicit Multi-Stepping prove effective in optimizing the numerical VOF scheme. Digital mapping using physical experiments and virtual simulations validates transient flow predictions, achieving excellent agreement with deviations as low as 1.48–3.34%. The accuracy of flow predictions is thereby greatly influenced by non-Newtonian viscosity characteristics in the low shear range and time-dependent contact angle variations. The study further explores flow manipulation concepts, focusing on local flow speed adjustment, gap segmentation, and the use of arcuate shapes to influence interface confluence near the chip. Experimental validation corroborates the effectiveness of each design intervention. In conclusion, this research highlights the potential of predictive engineering to develop flow-optimized package designs that enhance reliability while supporting high manufacturing yields.

## 1. Introduction

The integration of power electronics into compact modules has seen widespread adoption across various industries. As such, the advancement of electric vehicle inverters is driven by increasing requirements for power density and conversion efficiency, propelling a shift towards vertical integration as a viable alternative to conventional wire bonding approaches [1]. Traditional silicone-potted modules, featuring wire-bonded Si devices on ceramic substrates, grapple with concerns such as electrical parasitics, transient heat dissipation, and high-voltage isolation. To address these challenges, a range of planar interconnection techniques, including directly deposited copper, direct lead frame bonding, flexible PCB, or double-sided DBC, have emerged as wire bondless alternatives to enhance high-speed switching behavior and thermal management [2,3,4,5]. A great benefit of enclosing semiconductor devices between an upper and lower substrate is providing a secondary thermal path for improved heat dissipation [6,7,8,9,10]. Yet, this approach faces significant challenges, including the pronounced mismatch in the coefficient of thermal expansion (CTE) between Si/SiC chips and substrates, alongside stress-induced warping during high-temperature operations, predisposing fatigue failure. Moreover, as voltage levels increase, susceptibility to dielectric breakdown remains a pressing concern.

In response, the encapsulation of power modules assumes increasing importance to enhance dielectric, mechanical, and thermomechanical reliability. Present methodologies primarily entail transfer molding using package-specific molds with epoxy molding compounds (EMC). For this reason, recent advancements in compression molding technology have introduced a more flexible platform, enabling the encapsulation of diverse power packages via a single molding tool [11].

This article focuses on capillary underfilling as a versatile encapsulation technique, particularly suited to the intricate iterative development phases of novel vertical integration concepts. Unlike traditional approaches, this method eliminates the need for custom molds and subsequent processing steps. Nonetheless, ensuring a defect-free underfill application presents a critical challenge, given that both reliability and production throughput are significantly impacted by void formation and prolonged filling times [12]. Hence, it becomes imperative to not only tweak the material attributes of the underfill but also refine dispensing patterns and incorporate modifications in package designs to improve flow dynamics throughout the filling process.

In recent years, various studies have delved into the investigation of influential factors in underfill flow and the development of reliable models to predict capillary flow, albeit rarely in the field of power packaging [13]. Ng and Abas [12] conducted a comprehensive analysis spanning studies from 1996 to 2020, with a particular emphasis on visualizing and numerically modeling underfill flow. The review revealed that in the last few years, the landscape of numerical simulation methodologies for three-dimensional capillary underfill flow has witnessed considerable diversity. In addition to the traditional finite element method (FEM), the finite volume method (FVM) has emerged as a widely adopted approach for underfill simulations. Presenting a mesoscopic perspective, the Lattice–Boltzmann method (LBM) has gained attention, characterizing the behavior of filler particles through discrete lattice-based propagation and collision processes, rather than the direct solution of the Navier–Stokes equations [14]. Furthermore, simulations have extended their scope to encompass fluid–structure interaction (FSI) techniques that consider both fluid flow dynamics and structural deformations [15,16]. An instance of this is seen in transfer molding, where the rapid injection of EMC during the molding process can induce package deformation. However, in the realm of conventional capillary underfilling, substantial substrate and die deformations are not anticipated due to the relatively low flow velocities and marginal capillary forces.

Aside from the numerical methods used, the main focus of these studies was the geometry of the classic flip-chip packaging with gap height, bump shape, and pitch being the most studied parameters concerning design optimization [17,18,19,20,21]. Chen et al. recently proposed a digital twin approach, based on the Moldex3D IC packaging module, to study the filling outcome of a Flip-Chip Ball-Grid-Array (FCBGA) package for varying dispensing patterns [22]. However, the encapsulation of multi-chip power modules introduces new challenges such as varying gap heights and surfaces, intricate geometries with large-scale cavities, and particularly low capillary pressure gradients. Heterogenous material attributes, on the one hand, impede the tuning of underfill properties, while on the other, void occurrence may not always be mitigated by solely adjusting dispensing patterns. Therefore, further measures must be developed to tame the flow behavior.

This study applies the concept of a digital twin to the conventional underfilling of multi-chip power packages and presents a robust two-phase flow model as a basis for future package development. As an extended version, this paper covers two previous publications [23,24]. First, emphasis is put on the performance evaluations of two different modeling strategies, namely Level-Set and Volume of Fluid (VOF), as well as parametric optimization of the favored numerical scheme. Digital mapping of the physical experiment and virtual simulation are used to calibrate and verify the model, as well as to understand the root cause of frequently occurring macroscopic void formations. Second, exemplary design scenarios are explored, showcasing the model’s potential in generating custom flow-controlling modifications.

## 2. Materials and Experimental Methods

The following sections describe the materials used for physical experiments, material characterizations, and virtual mapping methods for experimental validation.

### 2.1. Vertical Integration Packaging Concept

**Figure 1:**
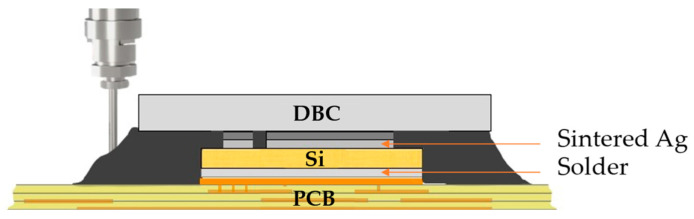
Schematic cross-section of the package structure for a single Si-IGBT.

This study primarily centers on the advancement of multi-chip half-bridge modules employing insulated gate bipolar transistors (IGBTs). These modules typically consist of pairs of 1200 V/600 A IGBTs along with a complementary free-wheeling diode, constituting a versatile multi-chip arrangement, as depicted in Figure 1. The semiconductor is situated between an organic substrate (FR4) and a ceramic substrate (Al_2_O_3_) and attached via solder joint (SnSb5) and a sintered Ag layer. Depending on the number of incorporated devices, the lateral dimensions can span up to 70 mm [6] with global gap heights between 200 and 600 µm. Separate connections for the gate and emitter of the semiconductor lead to local gap areas with gap heights lower than 80 µm. The in-plane shape of the sintered joint can thereby result in indentations highly prone to void formation. Furthermore, the presence of multiple chips or other gap-blocking elements may cause flow fragmentation and subsequent confluences that are hard to predict. The risk of void formation escalates as the number of chips increases.

The proposed simulation method must therefore account for locally varying wetting properties and widely varying gap heights. Beyond the specific use case, the model can also be employed for smaller gap heights of e.g., 10–30 µm to describe the capillary liquid spreading of viscous, non-dispersive fluids in microchannels in general. This opens up further potential applications in electronics packaging such as FCBGAs [22], as well as applications in microfluidics and lab-on-a-chip technologies [25,26,27].

### 2.2. Test Setup

To verify the transient simulation result with experimental flow recordings, an innovative strategy was introduced. It is worth noting that replacing the actual package substrates with transparent material dummies [28,29] changes the actual surface properties.

1. To retain a representative material composition, the flow was monitored using thinned FR4 and Al_2_O_3_ substrates. For die imitation, a copper layer was attached via solder/sinter preforms. Substrate materials and dimensions are listed in Table 1;
2. Test samples for flow calibration without any overhangs within the gap were illuminated from underneath to guarantee high-contrast images;
3. To include flow-optimizing design elements, dam underfill was applied before soldering. Benefits of this material include its limited wetting characteristics and its capacity to accommodate fluctuating gap dimensions;
4. After setup, the gap height of each specimen was measured using confocal laser scanning microscopy. Considering substrate warping caused by thermal expansion during the joining process, average gap heights were computed with polynomial fitting;
5. Translucency of the thinned substrates enabled real-time monitoring of the black-colored underfill, captured from a top view (xy-plane). Accordingly, this approach is well-suited for identifying voids across the entire gap height.

The underfill was thawed to room temperature one hour prior to the experiments, while the assembly was preheated to 60 °C. Thermocouples were used to control the temperature ensuring a uniform surface temperature. The underfill was applied on the bottom substrate along the edges of the upper substrate using a variable dispensing pattern. Once the gap was filled, the curing process was initiated at 135 °C.

### 2.3. Material Characterization

**Figure 2:**
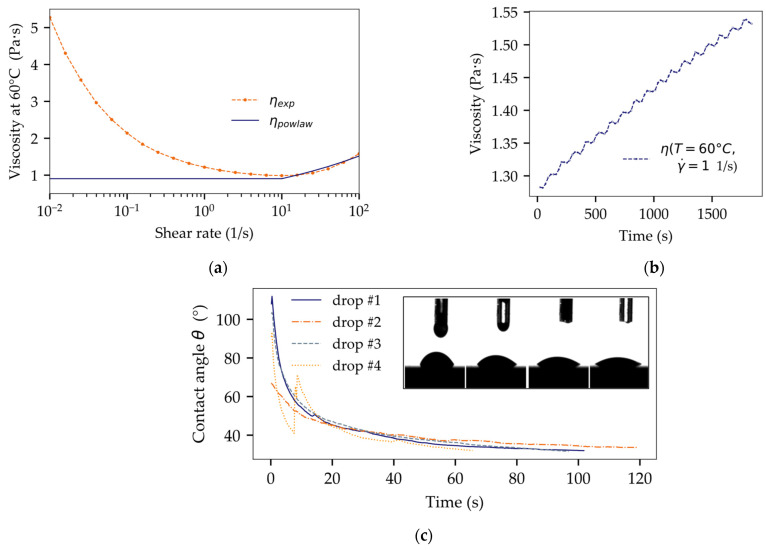
Characterization of material wetting behavior: (**a**) measured viscosity values and fitted power law at 60 °C; (**b**) gelling: time-dependent increase in viscosity at a constant shear rate of 1 1/s; (**c**) dynamic measurements of the contact angle by the example of underfill on FR4.

The viscoelastic characteristics of the underfill material were examined through shear-controlled viscosity measurements at the process temperature of 60 °C. The measurements were performed with a gap size of 500 µm on a MCR 302 rheometer, Anton Paar GmbH. The measured viscosity values were interpolated for a direct use in the simulation model. In addition, the measured data were also fitted with the commonly used power law using a constant viscosity for shear rates lower than 10 1/s. Simulation results for both approaches (Figure 2a) were compared to demonstrate how simplified model fitting can affect the accuracy of filling time predictions.

Moreover, time-dependent changes in viscosity were analyzed for isothermal conditions at 60 °C with a constant shear rate of 1/s, shown in Figure 2b. The results were used to describe a linear rate of underfill thickening due to exposure to air and premature material gelling (cross-linking) prior to the completion of the filling process.

Pendant drop and sessile drop methods at 60 °C (OCA 20, dataphysics) were adopted to measure the surface tension of the underfill and its contact angle on different solid surfaces, such as FR4, Al_2_O_3_, and Cu. Simulating the manufacturing process of considered packages, the samples were subjected to a sequential sinter and solder process prior to measurement. Each contact angle was analyzed over time to include wetting dynamics, as shown in Figure 2c, by defining a dynamic contact angle θ = f(t). The results of the material characterization are summarized in Table 2.

### 2.4. Virtual Mapping: Post-Processing of Flow Recordings

**Figure 3:**
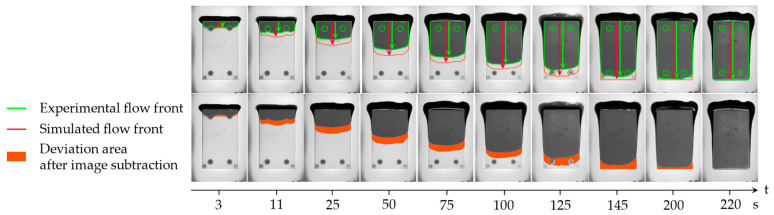
Example of post-processed image sequence for mapping physical experiment and transient numerical simulation data: binary masks of the filled gap area were used to plot the contour of experimental (green) and simulated interface (red). The central plane was used to evaluate the flow path length (green and red arrow, respectively). The lower row depicts the physical recording in top view overlayed with the extracted deviation within the gap (orange area).

The systematic evaluation of the deviations between numerical simulations and experimental results is illustrated in Figure 3. Both experimental and simulated video recordings were first transformed into a series of 2D grayscale images at one frame per second, using Matlab version R2022a [32]. These images were then consolidated into a 3D matrix and converted into a readable DICOM format within ITK-Snap 3.8.0 [33]. Once image alignment was assured, the DICOM sequences were standardized in terms of size and image resolution to ensure consistent voxel dimensions. For specific time points, distinct regions of interest (ROIs) were delineated to segment the volume filled with underfill material within the gap. Intermediate ROIs were generated using an interpolation function to capture time steps between chosen time points. By subtracting these two corresponding binary masks, the resulting area of deviation between the simulation and experiment was obtained over time, as depicted in Figure 3. The voxel count within this subtraction ROI, compared to a reference measure, was then translated into an absolute area measurement in mm² and normalized by the total gap area within the xy-plane. In addition, each of the binary masks was used to extract the central flow path length over time to depict filling dynamics.

## 3. Numerical Simulation

This study evaluates the performance of an FEM-based approach using COMSOL Multiphysics^®^ version 5.6 [34] compared to the FVM-based software Simcenter StarCCM+ version 2210 [35]. Within Comsol simulations, the Level-Set (LS) method was applied, while StarCCM+ simulations were implemented with the Volume of Fluid (VOF) approach to capture the interface between underfill and air.

### 3.1. Governing Equations

Both Eulerian multiphase methods treat the two phases as a unified continuous mixture of immiscible fluids governed by the same velocity field. In this study, the focus is set on epoxy-based underfill materials with elevated filler content and small particle sizes. Assuming a homogenous material was thus seen as a justifiable simplification to minimize computational costs and to make the simulation applicable to subsequent iterative design efforts. Given minimal capillary pressure gradients and Reynolds numbers, a laminar flow is assumed. Changes in underfill viscosity during the filling process are considered (Figure 2b); however, the model does not cover the subsequent curing phase.

The governing equations for both phases are represented by the continuity equation and the Navier–Stokes equation, which articulates the conservation of mass and momentum, respectively:

(1) $$\nabla \left(\rho u\right)=0\;\frac{\partial }{\partial t}\left(\rho u\right)+\rho \left(u\cdot \nabla \right)u=-\nabla p+\eta \nabla^{2}u+\rho g+f_{\mathrm{vol}}$$

The velocity and pressure fields (u,p) were solved through a coupled algorithm, while Level-Set/VOF methods were employed to track the shape and location of the interface separating underfill and air. To manage the two fluids within a unified framework (N = 2) using a one-fluid formulation, a volume fraction ϕ is defined for each control volume

(2) $$\phi_{i}=\frac{V_{i}}{V},\;\sum_{i=1}^{N}\phi_{i}=1$$

and relations are established to connect distinct material properties with ϕ:

(3) $$\rho =\phi \rho_{1}\;+\;1-\phi \rho_{2},\;\;\eta =\phi \eta_{1}+\;1-\phi \eta_{2}.$$

Here, ρ represents the density and η the dynamic viscosity. To track the advancing flow front, the transport equation

(4) $$\frac{\partial \phi }{\partial t}+u\cdot \nabla \phi =0$$

is resolved for the volume fraction of one of the two phases, designated as $\phi_{1}$. In this case, the primary phase is identified as air, while the secondary phase represents the underfill, expressed as $\phi_{2}$= 1 − $\phi_{1}$. The interface is defined by values of ϕ ranging between 0 and 1. The driving force of capillary action originates from the interfacial surface tension, which is a phenomenon that can be represented as a volumetric force employing Brackbill et al.’s continuum surface force (CSF) model [36]. This incorporates an additional factor into the momentum equation:

(5) $$f_{\mathrm{vol}}=\sigma \;\kappa n\;n=\nabla \phi ,\;\;\kappa =-\nabla \cdot \frac{\nabla \phi }{\left|\nabla \phi \right|},$$

where σ represents the surface tension, κ signifies the curvature of the free surface, and n denotes the vector perpendicular to the interface. To improve stability and mitigate spurious velocities at the interface, StarCCM + offers a semi-implicit surface tension model instead. In this approach, a time-based linear adjustment is included in the VOF simulation, which is directly linked to the surface tension and time step magnitude:

(6) $${\left(\kappa n\right)}^{n+1}\approx \underline{∆}x^{n}+∆t\underline{∆}u^{n+1},$$

with the Laplace–Beltrami operator $\underline{∆}$ and the identity mapping x on the free surface Γ. This modification counteracts parasitic currents by introducing an appropriate level of diffusion, thereby allowing for larger time steps.

### 3.2. Computational Domain and Boundary Conditions

Capillary pressure is driven by the interplay of contact angle θ, the underfill’s surface tension σ, and the distance h between parallel surfaces. This relationship is embodied in the Young–Laplace equation

(7) $$∆p=\frac{2\sigma \cos\theta }{h},$$

where ∆p represents the capillary pressure gradient across inlet and flow front. The ambient pressure acts on the inlet surfaces ($p=p_{\mathrm{atm}}$), which are defined in accordance with the underfill’s dispensing positions. In the forthcoming discussion, diverse modeling approaches are deliberated, each distinguished by its definition of the computational domain and boundary conditions. The aim is to strike a reasonable balance between precision and computational effort.

Previously, in classic flip-chip packaging (FCP), a common modeling strategy was to confine the computational domain to the gap volume between the chip and substrate. Figure 4① illustrates this approach, with pressure boundary conditions set to ambient pressure. Each surface within the gap is attributed its respective contact angle.

During the underfilling of FCBGAs, accelerated flow along the lateral chip edge was observed [28]. This phenomenon, known as the racing effect, manifests particularly when the distance between the outer solder balls and the chip edge widens and can lead to air entrapment due to uneven flow shapes [37,38]. However, this model approach is unsuited for FCPs with larger lateral pitches or for packages featuring expansive cavities, such as power packages. While the flow is well predicted in the gap’s central region, the model falls short in portraying lateral flow behavior [39]. A preliminary study with experimental flow data from a test package employing soldered FR4 and sputtered glass confirmed these findings for a gap height of 330 µm, see Figure 4①. To enhance the prediction of lateral flow, Zhu introduced alternative modeling methods that explicitly define the driving capillary pressure gradient at the outlets (Figure 4②,③). The gap’s solid surfaces are set as no-slip boundaries, and the outlet pressure gradient is determined via the Young–Laplace formulation (7). The second approach (Figure 4②) designates the lateral outlets as no-slip walls, while the third approach (Figure 4③) employs dynamic pressure boundary conditions linked to the current flow front position. Both methods prove inadequate for simulating flow behavior for complex dispensing paths other than I-shaped. Additionally, these methods rely on an averaged contact angle for all surfaces within the gap to compute the outlet pressure gradient. Hence, these techniques do not accommodate heterogeneous integration with varying surface characteristics. The simulation’s computational domain was therefore expanded to encompass the surrounding air at the gap’s edges, see Figure 4④. This approach avoids instabilities in peripheral gap regions and yields more realistic flow dynamics. It further includes the wetting of the lateral surface of the upper substrate, which has a significant influence on the lateral flow. As illustrated in Figure 4④, this model correlates well with the experimental flow.

### 3.3. Implementation of Varying Dispensing Patterns

**Figure 5:**
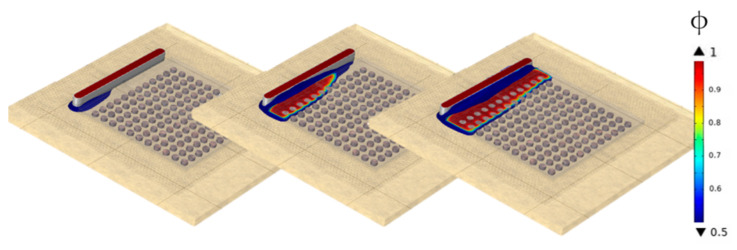
Definition of dynamic boundary conditions: inlet condition as a function of time and space if the ratio of speed to path length is small.

The model can be adjusted by introducing dynamic boundary conditions as functions of time and space. This is crucial for a dispensing path optimization when multiple dispensing positions are involved. It also becomes relevant if a single dispense line is extensive or the dispensing speed is low, resulting in an asymmetric flow front (Figure 5).

### 3.4. Coupling Two-Phase Flow with Heat Transfer

The dominant material properties governing the flow are notably sensitive to temperature variations. In cases where the assembly experiences significant temperature gradients during the filling process, a coupled two-phase flow simulation with heat transfer becomes advisable. Nonetheless, experimentation involving thermocouples and preliminary simulations of steady-state heat transfer have demonstrated minimal temperature gradients along the gap height, due to uniform preheating. Thus, the benefit of coupling heat transfer over assuming isothermal flow conditions is marginal in this case.

### 3.5. Grid Convergence Study

**Figure 6:**
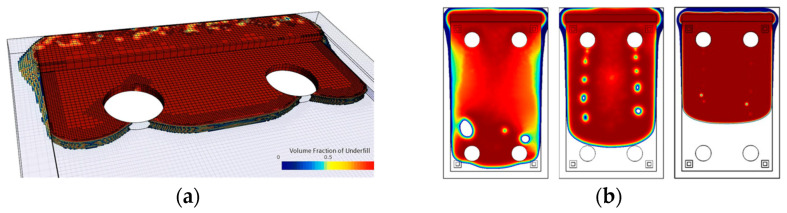
Constant mesh scaling within the region that is refined by AMR moving with the interface: (**a**) interfacial mesh refinement for f_2_; (**b**) Level-Set, from left to right: simulation results $f_{3}$, $f_{2}$, $f_{1}$.

To evaluate the performance of the two interface-capturing methods Level-Set and Volume of Fluid, a mesh convergency study was performed. The Grid Convergence Index (GCI) was used as a quantifiable and standardized measure to gauge spatial discretization errors [40]. A constant scaling factor, denoted as r = 2, was uniformly employed across all three spatial dimensions. Since the surface tension is the only driving force in the system and is modeled based on interface curvature, Adaptive Mesh Refinement (AMR) was employed to maintain a sufficient interface resolution throughout the process. Constant mesh scaling was carried out here by varying the maximum AMR level, see Figure 6a.

The filling time, which was defined as the time between initial dispensing and 90% coverage of the gap cavity, was calculated for a coarse ($f_{3}$), medium ($f_{2}$), and fine mesh ($f_{1}$) to determine the order of accuracy p:

(8) $$p=\ln\left(\frac{f_{3}-f_{2}}{f_{2}-f_{1}}\right)/\ln\left(r\right).$$

A recommended safety factor of $F_{s}$ = 1.25 was used to calculate the respective GCI:

(9) $$\mathrm{GC}I_{12}=\frac{F_{s}}{r^{p}-1}\left|\frac{f_{2}{-f}_{1}}{f_{1}}\right|,\;\;\mathrm{GC}I_{23}=\frac{F_{s}}{r^{p}-1}\left|\frac{f_{3}-f_{2}}{f_{2}}\right|.$$

An equality between $GCI_{12}$ and $GCI_{23}$ signifies asymptotic mesh convergence. Furthermore, the so called true numerical filling time (target value without any discretization errors), was estimated using the Richardson extrapolation method [41]:

(10) $$f_{h=0}≅f_{1}+\frac{f_{1}-f_{2}}{r^{p}-1}.$$

**Figure 7:**
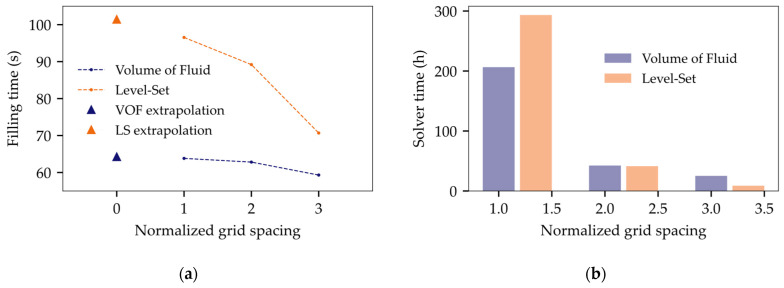
Illustration of grid convergence study: (**a**) convergence of filling time (target value) for constant mesh scaling; (**b**) required computation times depending on mesh refinement.

Following the outcomes presented in Table 3, it is evident that the Comsol solution $f_{1}$ did not achieve convergence. The fine mesh still deviates notably by 6.27% between the simulated filling time and the true value that is unaffected by spatial discretization errors, see Figure 7a. Yet, Level-Set requires more computational resources compared to VOF runtimes in Star-CCM+, as illustrated in Figure 7b. Apart from that, Level-Set reveals unphysical voids not seen in the experiment (Figure 6b).

Utilizing Comsol for further analysis would necessitate mesh refinement and therefore require even higher computational effort. The VOF model, in contrast, shows minimal discretization errors of 0.77% implying asymptotic convergence. Accordingly, VOF is considered superior to the Level-Set method in terms of efficiency and precision and is chosen for further analyses.

### 3.6. Parametric Optimization of VOF Model

The model faces considerable stability issues and extensive computation times due to limited capillary driving forces, extended filling durations, and pronounced interface discontinuities marked by significant variations in density and viscosity between underfill and air. This underscores the relevance of enhancing the chosen solver schemes to tailor the model to the specific use case requirements.

#### 3.6.1. Angle Factor, Blending Criterion, and HRIC Scheme

**Figure 8:**
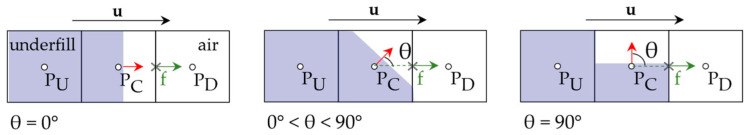
Interface orientation $\theta_{\mathrm{int}}$: angle between interface normal and cell face normal.

For VOF modeling, this is mainly carried out using a parametric optimization of the High-Resolution Interface-Capturing (HRIC) method [42]. The HRIC scheme addresses the advection term of the transport Equation (4) of the volume fraction ϕ [43]. Balancing precision and stability, this method combines a second-order downwind scheme (DDS) with an additional first-order upwind differencing scheme (UDS). As illustrated in Figure 8, $\theta_{\mathrm{int}}$ is defined as the angle between the interface normal and the cell face normal.

While DDS yields favorable outcomes for situations featuring an interface perpendicular to the flow direction ($\theta_{\mathrm{int}}$ = 0°), challenges arise when the phase boundary aligns parallel to the flow direction ($\theta_{\mathrm{int}}$ = 90°), potentially resulting in interface distortions and wrinkles. To correct such instabilities, a higher weighting of the additional upwind scheme is forced by an angle-dependent blending function:

(11) $$\xi_{f}^{*}={\left|\cos\theta_{\mathrm{int}}\right|}^{C_{\theta }}\xi_{f}+(1-|{\cos\theta_{\mathrm{int}}|}^{C_{\theta }})\xi_{C}.$$

**Figure 9:**
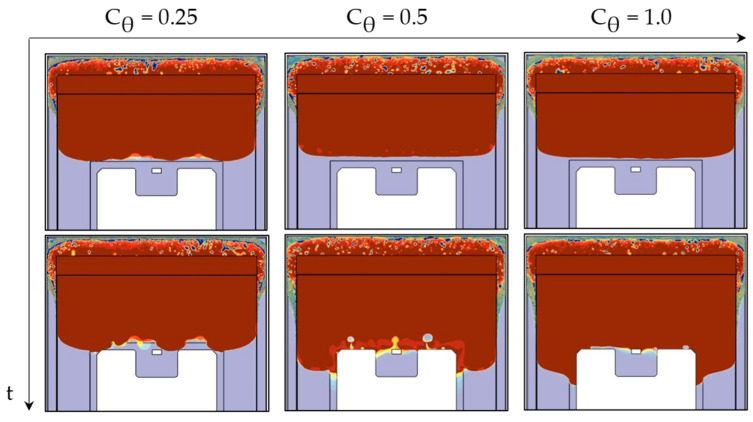
HRIC tuning through varying the angle factor to reduce interfacial distortions.

Adjustments to the blending function, governing the transition between DDS and UDS, can therefore significantly enhance interface resolution [43]. The influence of different angle factors $C_{\theta }$ was analyzed to determine the optimal HRIC blend criterion. As illustrated in Figure 9, the interface normal is tilted as soon as the flow approaches the die. To fill the overhang above the chip, the interface is briefly aligned parallel to the general flow direction causing severe instabilities. However, by increasing the angle factor up to $C_{\theta }$ = 1.0, the shape of the interface could be significantly stabilized and smoothed.

#### 3.6.2. Interfacial Artificial Viscosity

**Figure 10:**
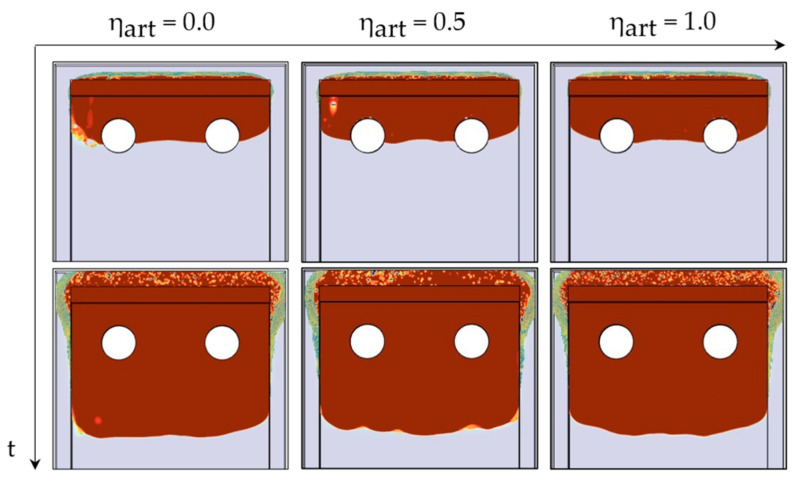
HRIC tuning through adding artificial viscosity to minimize to stabilize the interface.

StarCCM+ provides the option to introduce an artificial viscosity term to minimize spurious interfacial currents and smoothen interface dynamics [44]. Based on physical considerations, the system is expected to remain in a static equilibrium which means, ideally, the interface velocity is zero. Nevertheless, numerical simulations with strong discontinuities in material properties, usually introduce unphysical velocities at the interface, known as parasitic or spurious currents [45]. These velocities are mainly induced by discretization errors of the surface tension [46] and can distort the interface, as shown in Figure 10 for $\eta_{\mathrm{art}}$ = 0.0. As a result, discretization schemes may be stabilized by incorporating smoothing terms such as Laplacian filters [47]. By defining an Interface Momentum Dissipation Model, additional dispersive terms were added to the transport equation, damping out unwanted interfacial oscillations.

To assess the stabilizing impact of this additional artificial viscosity, the root mean square of the interface velocity magnitude $u_{RMS,t}$ was analyzed:

(12) $$u_{\mathrm{RMS},t}=\sqrt{\sum \left(u_{x,\mathrm{int},t}^{2}+u_{y,\mathrm{int},t}^{2}+u_{z,\mathrm{int},t}^{2}\right)/N_{\mathrm{cells},\mathrm{int},t}}.$$

**Figure 11:**
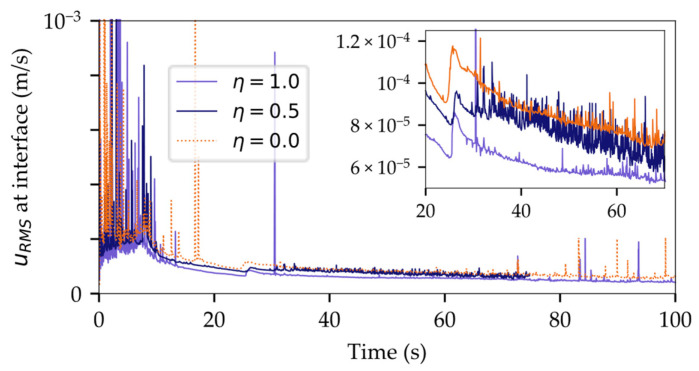
Successful reduction of spurious currents at the interface by increasing artificial viscosity.

As shown in Figure 11, applying an increasing artificial viscosity could effectively reduce the interfacial velocity magnitude corroborating the stabilizing effect in Figure 10.

#### 3.6.3. Implicit Multi-Stepping

The global time step, as determined by the Free Surface CFL Condition of the Adaptive Time Step (ATS) model, was split into three equal substeps for the VOF solver. This allowed increasing the CFL limits for the Segregated Flow solver, resulting in a much larger global time step size, while still maintaining a precise interface resolution through smaller time steps for interface propagation calculations.

**Figure 12:**
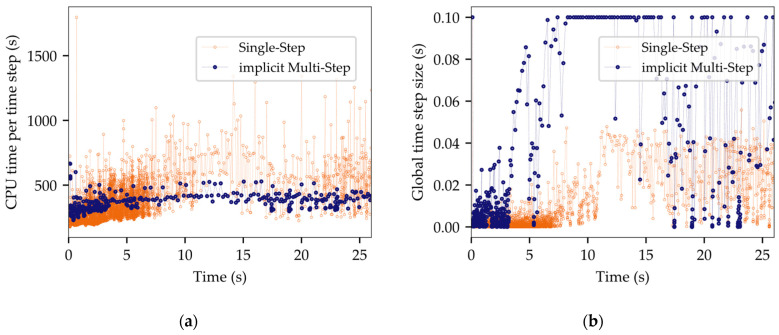
The benefit of introducing implicit Multi-Stepping: (**a**) comparable CPU times per time step; (**b**) sharp increase in global time step size for Multi-Stepping.

As depicted in Figure 12a, the CPU time per time step for both Single-Step and Multi-Step methods remained at a comparable level. However, the global time step size exhibited an increase by a factor of ~3–4, as shown in Figure 12b. Implicit Multi-Stepping is hence a viable strategy to curtail runtime without compromising performance.

## 4. Experimental and Numerical Results

### 4.1. VOF Sensitivity Study

**Figure 13:**
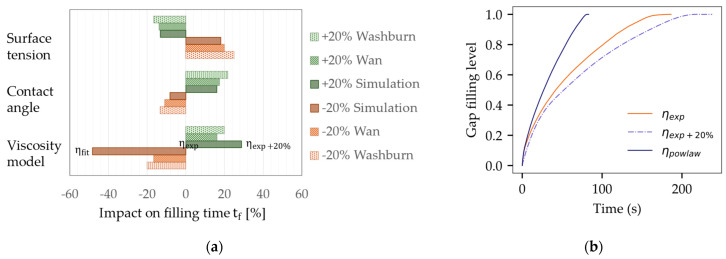
Sensitivity analyses for main factors influencing capillary flow behavior: (**a**) trends in the variation of input variables; (**b**) impact of varying viscosity definitions on computed filling time.

The computed filling time was selected as a target value to evaluate the impact of input parameter variations involving gap height, surface tension, contact angle, and viscosity within a range of ±20%. Figure 13a illustrates the simulation outcome, along with the corresponding changes predicted by two analytical models. The Washburn model represents the modeling of Newtonian fluid behavior [48]. Wan [49,50], by contrast, accounts for non-Newtonian behavior by means of a power law viscosity, but does not consider rapidly increasing viscosity values for very low shear rates, as observed in Figure 2a.

In addition, modified analytical models, as that of Darbois Texier et al. [51], were developed to assess the impact of different micropillar arrays on the flow dynamics. This Washburn-based model can predict filling times, e.g., for FCBGAs with high pillar densities, but only accounts for Newtonian behavior.

Figure 13a shows a fair agreement between the simulation and analytical predictions, although the Washburn and Wan predictions are notably less sensitive to viscosity changes. Figure 13b illustrates the effect of different viscosity models. The reference simulation was performed with interpolated measurement data $\eta_{\exp}$. A shift of +20% led to an increase in the filling time of 28.9%. Instead of a reduced viscosity, the power law fit was used, as this method was a common strategy in previous publications [52,53,54,55]. However, the simulation based on $\eta_{\mathrm{fit}}$ indicated flow velocities twice as fast as those calculated with $\eta_{\exp}$. This observation underlines the significance of capturing low shear viscosity changes to obtain an accurate projection of the flow behavior. The premise of a fixed viscosity $\eta_{0}$ for low shear rates was found to be unsuitable for the underfill material employed in this study.

### 4.2. Experimental Verification

**Figure 14:**
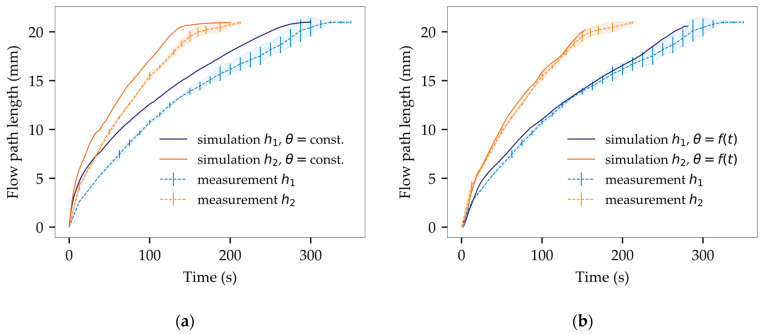
Analysis of transient flow path length for experimental flow versus simulation: (**a**) temporal offset mainly due to constant contact angles; (**b**) successful alignment of simulation results within experimental deviations through the definition of dynamic contact angles.

A simplified geometry was used to assess the flow velocity for varying gap heights ($h_{1}$ = 214 µm, $h_{2}$ = 430 µm). As shown in Figure 3, preliminary simulation outcomes exhibit favorable agreement with the corresponding experimental flow in terms of flow pattern. Nevertheless, a temporal deviation can be seen as the simulation is strongly preceding at the beginning of the filling process. In the medium term, flow velocity of simulation and experiment converges, leaving a constant offset between both flow patterns (Figure 14).

As depicted in Figure 14a, test samples with reduced gap heights displayed slower filling times, aligning with theoretical expectations and previous literature [56]. The measured average filling times ($t_{f1,\exp}$ = 334 s, $t_{f2,\exp}$ = 212 s) were compared to solutions based on the Wan model. Analytically derived values ($t_{f1,\mathrm{Wan}}$ = 180 s, $t_{f2,\mathrm{Wan}}$ = 90 s) significantly diverged from experimental findings, underscoring the benefit of numerical simulation.

The deviation of the initial flow velocity was further investigated, and three hypotheses were put forward to account for the temporal offset:

1. Simplified inlet condition: simulation assumes complete wetting of the gap, whereas several seconds are necessary for adequate material deposition;
2. Heat transfer within underfill is neglected: since the underfill was not preheated before application, it undergoes a temperature transition from room temperature to 60 °C, resulting in a lower initial experimental flow velocity;
3. Time-dependency of the contact angle θ(t): in alignment with time-dependent sessile drop measurements (Figure 2c), the equilibrium contact angle is reached not before 60 s. This dynamic aspect was not considered in the previous model, contributing to temporal discrepancies.

Following this, the model parameters were adjusted and are specified in Table 4.

**Figure 15:**
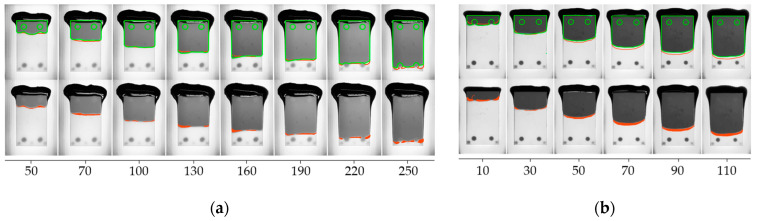
Experimental verification of adapted model after introducing dynamic contact angles. The physical recording is depicted in top view, overlayed with the contour of experimental (green) and simulated interface (red) and the extracted deviation within the gap (orange area): (**a**) exemplary sample for $h_{1}$ = 214 µm; (**b**) $h_{2}$ = 430 µm.

As depicted in Figure 15, the use of dynamic contact angles has significantly reduced deviations between the simulation and the experiment. The normalized area between the simulated and the experimental interface reveals average deviations of 1.48% for $h_{1}$ and 3.04% for $h_{2}$. Considering the variation of empirical flow data, the simulation now reveals a successful match with an accurate representation of both flow pattern and flow velocity.

Subsequently, the validated VOF model was employed to evaluate more intricate geometries and further validate the computed outcomes.

**Figure 16:**
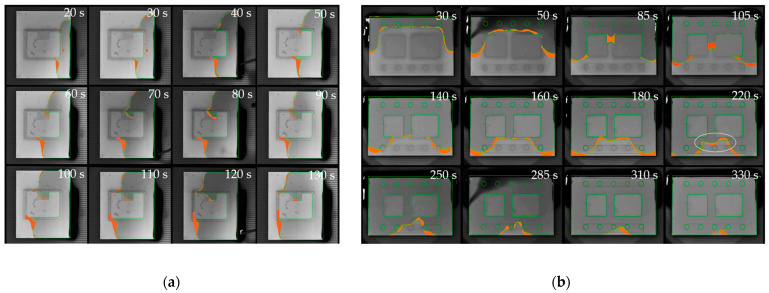
Overlay of experimental flow (green) and simulated interface with corresponding deviation area (orange): (**a**) U-shaped interconnection of single Si-MOSFET; (**b**) Multi-Chip Module.

1. Figure 16a illustrates a filling scenario of a single IGBT chip with emitter and gate pad being mounted on a ceramic substrate. The model accurately replicated critical flow behavior near the gate interconnection with a low average deviation of only 2.55%. The deep indentation of the sintered joint and the resulting passage around the gate do not allow for void-free filling and inevitably lead to air being trapped between emitter and gate. The simulation has convincingly replicated this behavior.
2. In Figure 16b, a common scenario is depicted where the geometric configuration results in irregularly shaped flow fronts, giving rise to uncontrolled merging of multiple free surfaces and, consequently, an increased likelihood of air entrapment. Once again, the simulation showed impressive agreement with the experimental results (2.84% deviation). Minor deviations of the interface primarily resulted from shape deviations and indentations in the joining zones within the experimental setup. Meniscus-shaped solder joints and areas of non-densified, porous sintering material seem to have an impact on the flow along the lateral chip surfaces.

## 5. Design Studies for Targeted Flow Manipulation

This study contributes to a deeper comprehension of the interplay between capillary flow and package design, shedding light on strategies for manipulating the flow and enhancing encapsulation outcomes. One possible approach is using the model to pinpoint optimal dispensing patterns. Nevertheless, intricate Multi-Chip Modules might not achieve void-free encapsulation solely by altering the dispensing pattern. Beyond specific applications, another pathway is to explore general design features that could be integrated into future power package designs to control the flow and mitigate voiding risks. This section proceeds to categorize flow manipulation measures into three possible strategies.

### 5.1. Flow Speed Adjustment

Both increasing gap heights and decreasing contact angles were shown to elevate flow velocities [57,58,59]. However, these parameters can also be harnessed as design features to locally manipulate the flow pattern.

**Figure 17:**
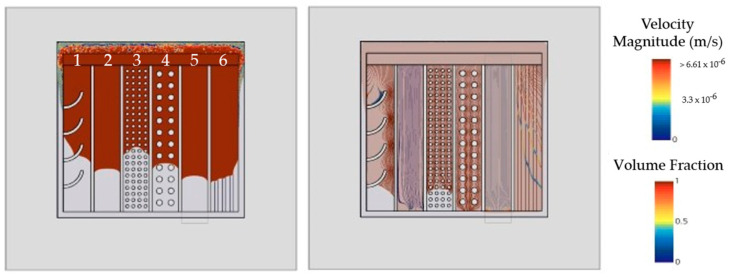
Design study to discuss general strategies for local speed adaption.

Figure 17 depicts a generic gap configuration between two substrates at 300 µm height with θ = 30°. The gap is divided into six channels, each filled with distinct flow barriers to compare elements in terms of flow acceleration and deceleration.

1. Arch-shaped flow barriers decelerate and redirect the flow, tilting the interface orientation towards the outer periphery. This mechanism can also transport unavoidable voids to a less critical peripheral gap area;
2. A flat layer (100 µm) was introduced to slow down the flow by reducing the cross-sectional height. Vice versa, to intentionally accelerate the flow, the inclusion of non-functional metallization areas with easily wettable surfaces is an option. Note that any additional layer will reduce gap height, weakening the accelerating effect;
3. Solder bump arrays may also be used for flow manipulation. Squared instead of cylindrical pillars [51] and smaller pitches lengthen filling times but also increase the risk of void occurrence [17,18,19,20,21], which is potentially unfavorable for encapsulation outcomes;
4. Higher bump pitch for more manageable flow behavior;
5. Stands as a reference flow without any modifications;
6. Additional layers for lowering the contact angle are more efficient, if incorporated in a striped shape instead of full coverage. This can be beneficial when substrate depressions are not a viable option to increase the local gap height.

### 5.2. Gap Compartmentation

**Figure 18:**
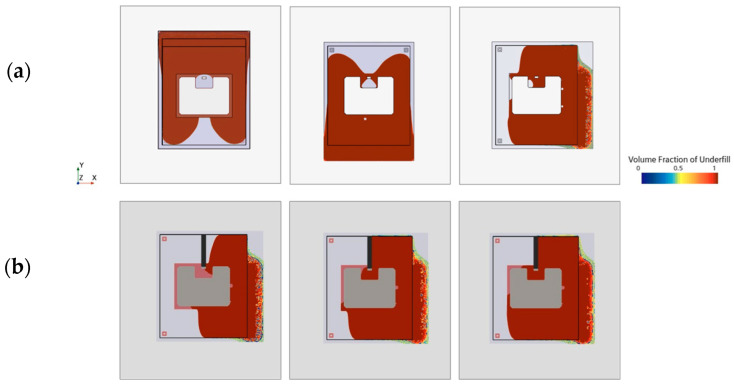
Different strategies to address void formation: (**a**) attempt to optimize length and location of dispensing path; (**b**) successful void prevention through gap compartmentation.

In areas susceptible to voids, design elements can obstruct the flow to prevent unwanted confluences. Pockets, which do not allow for simultaneous inflow and outflow of the underfill (dead ends), can be split into curved channels. Figure 18 displays the test sample of a single IGBT with U-shaped top side interconnection. Figure 18a shows an I-shaped dispensing pattern along the upper substrate edge that causes the horizontal flow to trap air while entering the indentation. Exploring various flow angles through adjustments in the dispensing position produces more favorable outcomes (Figure 18a). However, air entrapment still occurs within the lower left corner. Apart from altering the shape of the interconnecting layer, employing design elements to obstruct and direct the flow can serve as an alternative approach, as depicted in Figure 18b.

### 5.3. Enforcing Chip-Near Confluence

**Figure 19:**
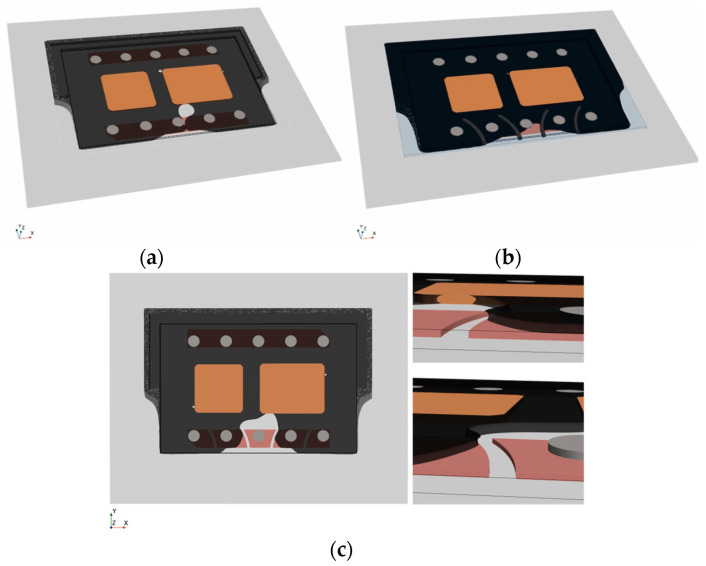
Impact of arched design elements: (**a**) void-critical Dual-Chip Module; (**b**) successful void prevention through incorporating valve-like design elements; (**c**) advanced integration concept.

Dual-Chip module-inspired geometries, as illustrated in Figure 16b, become critical when material compositions are changed, see Figure 19a. Flow blockage by two devices perpendicular to the flow results in three colliding faces. The confluence of multiple flow fronts generally raises the risk of air entrapment near the die. Dispensing path optimization can prevent void formation but is also likely to result in complex dispensing patterns that lengthen the filling process and reduce production throughput.

Drawing inspiration from biological vein valves, Figure 19b introduces arch-shaped elements wedged between the substrates. These valve-like structures disrupt parabolic flow fronts, wherein the curved shape is crucial for slanting the interface normal. The tilted orientation causes the right and left interface to meet very close to the die. By dividing the flow into separate compartments, the underfill is directed along the flaps towards the periphery, effectively preventing void formation in critical areas.

Figure 19c presents an adapted design variant in which valve-like structures are integrated into the PCB design through recesses in the top metallization layer. This showcases a method of incorporating abstract design elements without introducing additional material into the gap. As demonstrated in Figure 19c, the mechanism in this case is based on a combination of abrupt height changes due to sharp 90° edges, flow deflection, and deceleration.

### 5.4. Experimental Validation of Design Element Viability

The previous chapters were intended to indicate the potential of using the simulation model as a design tool driving innovation to come up with novel ideas for flow-optimized designs. The derived concepts were finally tested for their effectiveness using the established validation approach.

**Figure 20:**
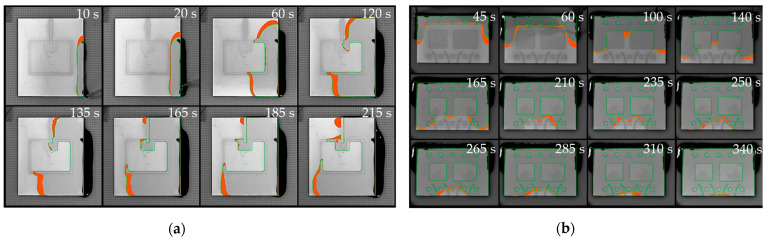
Experimental verification of design elements with experimental interface (green) and the deviation to the simulated interface (orange area): (**a**) comparted channel through U-shaped interconnection of single Si-MOSFET; (**b**) Multi-Chip Module with arched elements.

1. The results in Figure 20a confirm successful flow control by routing the underfill through the gate channel without air entrapment. Below the semiconductor, the experimental flow propagates slightly faster than the simulation because of non-planarity occurring during assembly. The rise in temperature during soldering triggers the shrinkage of the dam material, leading to substrate tilting, which was not included in the geometry model. Discrepancies between the experiment and the simulation still demonstrate good agreement with only 3.34% deviation.
2. Figure 20b highlights the benefit of arcuate valve-like features and confirms, once again, the accuracy of the simulation (2.84% deviation). The numerical as well as the experimental results demonstrated successful void prevention for both fully obstructing and half-height dam elements. Based on this finding, flow control measures do not necessarily need to adopt constricting dam designs. The redirection of flow can also be achieved through partial gap blockage or sharply designed recesses, offering a viable alternative without adverse consequences like substrate tilting or warping.

**Figure 21:**
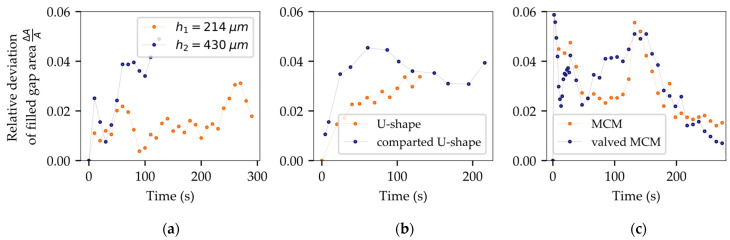
Overview of assessed deviations for all tested designs: (**a**) test samples for flow calibration with varying gap heights; (**b**) IGBT with U-shaped connection of the emitter, with and without obstructing elements; (**c**) Multi-Chip Module (MCM) with and without valve-like elements.

Figure 21 summarizes the results of the virtual mapping for all tested sample types. The time-averaged deviation for each design is depicted in Table 5.

## 6. Conclusions

In the context of encapsulating planar power packages, a numerical framework for capillary underfilling was successfully implemented, utilizing both Level-Set (Comsol) and Volume of Fluid (Star-CCM+) methods. Performance evaluation has favored VOF, providing more advanced capabilities for model calibration tailored to the demands of specific applications. Improvements in precision, stability, and computational efficiency were successfully achieved by optimizing the HRIC scheme. This involved making adjustments in discretization blending, incorporating additional dissipation terms, and implementing implicit Multi-Stepping. The tuned VOF model exhibits excellent accuracy in predicting flow patterns and flow velocities, validated through various experimental trials, showing deviations as minor as 1.48% to 3.34%. Moreover, geometries mimicking application-based designs were tested, showcasing the effectiveness of customized design features for flow manipulation.

Backed by a robust simulation model, alongside effective validation strategies, this research provides a solid foundation for future studies in capillary underfilling design and process optimization. These findings hold significant implications for semiconductor packaging, advanced integrated circuits, and sensors, where void-free underfilling is critical for device reliability and performance. Beyond reactive troubleshooting, the simulation has evolved into a proactive and predictive tool that seamlessly integrates into the development workflow, encouraging early consideration of flow optimization strategies.

On a broader scale, the proposed simulation method has versatile applications in modeling the wicking in microchannels between two plates, making it a valuable tool, for example, in microfluidics and lab-on-chip technologies relevant to healthcare, diagnostics, and chemical analysis.

## Figures and Tables

**Figure 4:**
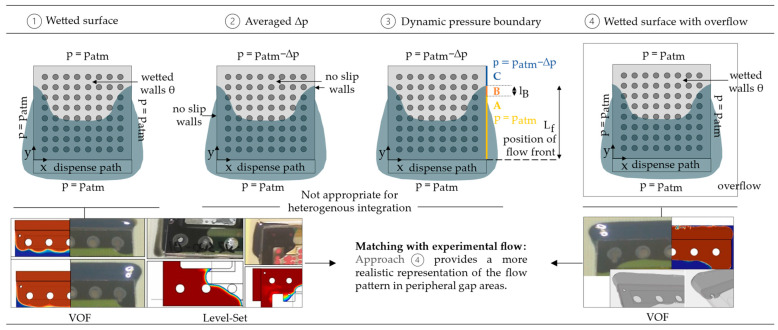
Evaluation of different modeling strategies aiming at minimized computational effort.

**Table 1:** Materials and dimensions of different test assemblies.

Flow Calibration		IGBTs		Multi-Chip Module	
FR4	800 µm	FR4	800 µm	FR4	800 µm
Al_2_O_3_	360 µm	Al_2_O_3_	360 µm	Al_2_O_3_	360 µm
-	-	Cu	120 µm	-	-
$\mathrm{SnSb}5\;h_{1}$	214 µm	SnSb5	110 µm	SnSb5	110 µm
$\mathrm{SnSb}5\;h_{2}$	430 µm	Sintered Ag	80 µm		
12.7 mm × 21 mm ^1^		21 mm × 25.4 mm ^1^		25.4 mm × 38.6 mm ^1^	

^1^ Dimensions of filled gap, determined by the size of the upper Al2O3 substrate.

**Table 2:** Material properties of underfill and solid surfaces used as simulation input parameters.

**Filler Type**	**Filler Content (%)**	**Max Particle (µm)**	**Density (kg/m³)**	
Quartz	67	1.5	1730	
**Viscosity**	**Power law fit**			
$\eta \left(\dot{\gamma },T\right)$	m (Pa·s)	n (1)	${\dot{\gamma }}_{0}$ (1/s)	${\dot{\gamma }}_{\infty }$ (1/s)
(see Figure 2a)	0.54	1.22	10	100
Gelling effect at 60 °C, $\dot{\gamma }$ =1 1/s (mPa·s):		$\eta \left(t\right)\;=1287.2\;\mathrm{mPa}\cdot s+0.142\;\mathrm{mPa}\cdot t$		
**Surface Tension at 60 °C**	**(mN/m)**			
SFT	Dispersive	Polar		
25	24.52	0.22		
**Contact angle at 60 °C (°)**				
FR4	Al_2_O_3_	Cu	SnSb5	Ag
35	25	33	30 [30]	25 [31]

**Table 3:** Grid convergence study using the GCI as a benchmark for performance evaluation.

Level-Set						VOF					
r = 2	AMR	$t_{f}$ (s)	p	$f_{h=0}$ (s)	$t_{\mathrm{solve}}$ (h)	r = 2	AMR	$t_{f}$ (s)	p	$f_{h=0}$ (s)	$t_{\mathrm{solve}}$ (h)
$f_{1}$	2	96.55	1.33	101.39	293.41	$f_{1}$	4	63.82	1.82	64.21	206.48
$f_{2}$	1	89.20			41.38	$f_{2}$	3	62.82			42.43
$f_{3}$	0	70.69			8.85	$f_{3}$	2	59.31			25.22
$\mathrm{GC}I_{12}$	6.27%	$\frac{\mathrm{GC}I_{23}\;}{\mathrm{GC}I_{12}}=1.08\neq 1$				$\mathrm{GC}I_{12}$	0.77%		$\frac{\mathrm{GC}I_{23}}{\mathrm{GC}I_{12}}=1.016≅1$		
$\mathrm{GC}I_{23}$	17.08%					$\mathrm{GC}I_{23}$	2.76%				

**Table 4:** Model parameter adaption for stabilizing interface and reducing temporal deviations.

Continua Model	Parameter	Previous Model	Modified Model
AMR	Max. Refinement Level	3	3
	Transition Width	6	6
	Delta Time (s)	0.1	0.1
ATS	Max. CFL Limit	0.4	1.0
Multiphase Interaction	Semi-implicit Surface Tension	enabled	Enabled
	Interface Artificial Viscosity	0.1	1.0
HRIC	$\mathrm{CF}L_{l}$	0.5	0.5
	$\mathrm{CF}L_{u}$	1.0	1.0
	Sharpening Factor	0.0	0.1
	Angle Factor	0.05	1.0
Segregated Flow	Enhanced Stability Treatment	enabled	enabled
	URF Velocity	0.8	0.8
	URF Pressure	0.2	0.2
Segregated VOF	Solution Strategy	Single-Step	Multi-Step
	URF VOF	0.9	0.9
	Number of Steps	1	3

**Table 5:** List of averaged deviations between physical experiment and numerical simulation.

Flow Calibration		IGBTs		Multi-Chip Module	
$h_{1}$ = 214 µm	1.48%	U-shape	2.55%	Dual-Chip	2.84%
$h_{2}$ = 430 µm	3.04%	Comparted	3.34%	Valved MCM	2.79%

## Data Availability

The data supporting the reported results are available on request from the corresponding author.

## Nomenclature

**List of symbols:**			**List of abbreviations:**	
η	Pa·s	Dynamic viscosity	AMR	Adaptive Mesh Refinement
$\eta_{\exp}$	Pa·s	Interpolated measurement viscosity data	ATS	Adaptive Time Step model
$\eta_{\mathrm{powlaw}}$	Pa·s	Fitted power law viscosity	CFD	Computational Fluid Dynamics
$\eta_{\mathrm{art}}$	Pa·s	Interfacial artificial viscosity	CFL	Courant–Friedrichs–Lewy condition
$\eta_{0}$	Pa·s	Fixed initial viscosity for low shear rates	CTE	Coefficient of Thermal Expansion
m	Pa·s	Flow consistency index (power law)	DBC	Direct Bonded Copper
n	1	Flow behavior index (power law)	DDS	Differential Downwind Scheme
$\dot{\gamma_{0}}$	1/s	Zero shear rate limit of power law definition	DICOM	Digital Imaging and Communications in Medicine
$\dot{\gamma_{\text{∞}}}$	1/s	Asymptotic shear rate limit of power law	EMC	Epoxy Molding Compound
θ	°	Contact angle	FCBGA	Flip-Chip Ball-Grid-Array
u	m/s	Velocity field	FCP	Flip-Chip Package
p	Pa	Pressure field	FEM	Finite Element Method
$\phi_{1}$	1	Volume Fraction of underfill	FSI	Fluid–Structure Interaction
$\phi_{2}$	1	Volume Fraction of air	FVM	Finite Volume Method
ρ	kg/m³	Mass density	GIC	Grid Convergence Index
g	m/s²	Gravity	HRIC	High-Resolution Interface-Capturing scheme
$f_{\mathrm{vol}}$	N/m	Volumetric force	IGBT	Insulated-Gate Bipolar Transistor
σ	N/m	Surface tension	LBM	Lattice–Boltzmann Method
n	1	Interface normal	LS	Level-Set
κ	1	Interface curvature of the free surface	MCM	Multi-Chip Module
$\underline{\text{∆}}x$	1	Laplace–Beltrami operator of identity mapping	PCB	Printed Circuit Board
Γ	1	Free surface	ROI	Region of Interest
∆p	Pa	Young–Laplace capillary pressure gradient	SFT	Free Surface Tension
h	m	Gap height	Si/SiC	Silicon/Silicon Carbide
$p_{\mathrm{atm}}$	Pa	Atmospheric pressure	MOSFET	Metal-Oxide-Semiconductor Field-Effect Transistor
R	1	Mesh scaling factor	UDS	Upwind Differential Scheme
p	1	Order of accuracy for GCI calculation	URF	Under-Relaxation Factor
$F_{s}$	1	Safety factor (1.25 for three iterations)	VOF	Volume of Fluid
$f_{i}$	-	Simulated target value for $i^{\mathrm{th}}$ mesh iteration		
$f_{h=0}$	-	True numerical target value		
$t_{f}$	s	Filling time for 90% gap coverage		
$t_{\mathrm{solve}}$	h	Required solver time		
$\theta_{\mathrm{int}}$	°	Angle between interface and cell face normal		
$C_{\theta }$	1	Angle factor of HRIC blending criterion		
$\xi_{f}^{*}$	1	Blended normalized cell face value		
$u_{RMS,t}$	m/s	Root mean square of interface velocity magnitude for time t		
$u_{\mathrm{xyz},\mathrm{int},t}$	m/s	Interfacial velocity component in x, y, z		
$N_{\mathrm{cells},\mathrm{int},t}$	1	Number of cells at interface		
